# Genetic relationships between spring emergence, canopy phenology, and biomass yield increase the accuracy of genomic prediction in *Miscanthus*

**DOI:** 10.1093/jxb/erx339

**Published:** 2017-10-12

**Authors:** Christopher L Davey, Paul Robson, Sarah Hawkins, Kerrie Farrar, John C Clifton-Brown, Iain S Donnison, Gancho T Slavov

**Affiliations:** 1 Institute of Biological, Environmental and Rural Sciences, Aberystwyth University, Aberystwyth, UK; 2 Computational and Analytical Sciences Department, Rothamsted Research, Harpenden, UK

**Keywords:** Bioenergy crops, biomass yield, breeding, canopy phenology, emergence, genomic prediction, genomics, genomic selection, *Miscanthus*, quantitative genetics, selection indices

## Abstract

*Miscanthus* has potential as a bioenergy crop but the rapid development of high-yielding varieties is challenging. Previous studies have suggested that phenology and canopy height are important determinants of biomass yield. Furthermore, while genome-wide prediction was effective for a broad range of traits, the predictive ability for yield was very low. We therefore developed models clarifying the genetic associations between spring emergence, consequent canopy phenology and dry biomass yield. The timing of emergence was a moderately strong predictor of early-season elongation growth (genetic correlation >0.5), but less so for growth later in the season and for the final yield (genetic correlation <0.1). In contrast, early-season canopy height was consistently more informative than emergence for predicting biomass yield across datasets for two species in *Miscanthus* and two growing seasons. We used the associations uncovered through these models to develop selection indices that are expected to increase the response to selection for yield by as much as 21% and improve the performance of genome-wide prediction by an order of magnitude. This multivariate approach could have an immediate impact in operational breeding programmes, as well as enable the integration of crop growth models and genome-wide prediction.

## Teaser

Multivariate analysis unveils complex genetic relationships between spring emergence, canopy phenology and biomass yield in *Miscanthus*, leading to increased response to selection and substantial improvements in genome-wide prediction.

## Introduction

*Miscanthus* is a perennial C_4_ grass that has great potential as a bioenergy crop (Visser and Pignatelli, 2001; Hastings *et al.*, 2009; Zub and Brancourt-Hulmel, 2010; Somerville *et al.*, 2010) to be used as part of the strategy for carbon mitigation and the control of climate change (Clifton-Brown *et al.*, 2004). Its above-ground biomass can be used both as a source of combustible material and as a substrate for liquid fuel production and other renewable materials. *Miscanthus* can produce high yields (Beale and Long, 1995; Clifton-Brown *et al.*, 2001, 2004) with low agricultural inputs across an extensive geographic range (Clifton-Brown *et al.*, 2007; Hastings *et al.*, 2009). In addition, with increasing concerns over food security, its ability to grow on marginal land not cultivated for food production will take on increasing significance.

At its simplest the biomass yield of a plant can be considered the product of the amount of photosynthetically active radiation (PAR) it receives over the growing season, the proportion of that radiation it intercepts and the efficiency with which it uses the intercepted PAR to create dry matter (radiation use efficiency; RUE; Monteith, 1977). Beale and Long (1995) considered several processes that contribute towards photosynthetic carbon fixation and concluded that light interception had the greatest potential to increase yield in perennial C_4_ grasses. The amount of PAR the plant intercepts is intimately related to canopy phenology, both in terms of canopy duration from emergence to senescence and in relation to the area of leaves deployed to intercept the light (leaf area index; LAI) over time (Hastings *et al.*, 2009; Davey *et al.*, 2016). Consistent with this model, Olson *et al.* (2012) showed that the high biomass yield of an energy sorghum hybrid was due to its extended period of growth, its high LAI, good PAR interception and high RUE. Similarly, Madakadze *et al.* (1998) found that the high biomass yield of cordgrass growing in a Canadian trial was due to its early spring growth and its ability to carry on growing into the autumn, thus maximizing its light interception over the growing season. However, the relative importance of phenology may depend on environmental conditions. For example, Zub *et al.* (2012) suggested from their studies of *Miscanthus* growing in northern France that the emphasis of breeding for increased biomass in this crop should be on increased growth rate and not on canopy duration, with the best performing genotypes having late spring emergence times. In contrast, trials of *Miscanthus* growing at the slightly higher latitudes of Wales (UK) demonstrated the importance of extended canopy duration for higher yield, with early emergence being slightly more important than extended growth at the end of the season (Robson *et al.*, 2013*a*). Early production of a light-intercepting canopy was also found to be important for increasing yield in mathematical modelling studies of four *Miscanthus* genotypes grown in Wales (Davey *et al.*, 2016). Finally, the length of the vegetative growth phase of a crop and hence its potential to generate biomass is also controlled by the initiation of flowering. Late flowering correlates with increased biomass in *M. sacchariflorus* (Jensen *et al.*, 2013), and selection for late flowering was instrumental in the development of energy sorghum varieties (Mullet *et al.*, 2014). Thus, while the interrelations between phenology and biomass yield have not yet been studied extensively in C_4_ grasses (Sarath *et al.*, 2014), they are likely to have nuanced genetic and an environmental context.

*Miscanthus* is an undomesticated crop, and the mechanistic and genetic basis of its biomass yield production are still relatively poorly understood (Davey *et al.*, 2016), making predictions challenging. This is particularly the case for genotypes other than the high yielding commercial *Miscanthus×giganteus*, a natural hybrid of *Miscanthus sinensis* and *Miscanthus sacchariflorus* (Greef and Deuter, 1993). In broad collections of these two species, canopy height appeared to be the best single predictor of biomass at both the individual plant (Robson *et al.*, 2013*b*) and genotype levels (Slavov *et al.*, 2014). However, although genome-wide prediction (i.e. trait prediction using a genome-wide set of molecular markers, also known as genomic selection) was effective for a broad range of traits related to phenology, cell wall characteristics and biomass productivity (i.e. including stem height/diameter/number and canopy height), the predictive ability for biomass yield *per se* was very low (Slavov *et al.*, 2014).

The first objective of this study was to clarify the genetic association between spring emergence and consequent canopy phenology, as well as determine how these traits affect the dry matter yield harvested in the following spring (Lewandowski and Heinz, 2003), accounting for the influence of other morphological and phenological traits. More importantly, our second objective was to assess if exploiting these relationships could improve genome-wide prediction of biomass yield, as well as to develop pragmatic multivariate approaches that could easily be implemented in breeding programmes.

## Materials and methods

### Phenotypic and molecular marker data

The phenotypic trait data used in this study were obtained from a field trial planted in 2005 near Aberystwyth, Wales, UK (52.432, −4.019). A detailed description of the trial was given by Allison *et al.* (2011), Jensen *et al.* (2011) and Robson *et al.* (2012). Briefly, 244 *Miscanthus* genotypes were planted at 1.5 m×1.5 m spacing in a randomized complete block design with four blocks, each containing one replicate of each genotype. A subset of 138 *M. sinensis* genotypes was defined as a genetically homogeneous population based on molecular marker analyses in two previous studies (Slavov *et al.*, 2013, 2014). In addition, 30 genotypes that had been classified as *M. sacchariflorus* based on molecular markers (Slavov *et al.*, 2013) were included for comparison. Plants were phenotyped in 2008 and 2009 for the traits listed in Table 1 using the methods described by Slavov *et al.* (2013, 2014). In addition, for each plant the highest emergence score was recorded on a given day in the spring. The scoring was as follows: 0, no emergence; 1, emergence from the soil as new shoots; 2, small shoots (<10 cm) from over wintered or new growth; 3, elongation of new growth or over wintered bud as a shoot; 4, leaf emergence; and 5, the shoot exceeds 10 cm in length. The dates on which genotypes first reached each emergence score were also estimated using linear interpolation. All phenological and biomass-related traits were measured for both species in both years to allow valid cross-comparisons of the data.

**Table 1.** *Phenotypic traits measured in 2008 and 2009 in two species of* Miscanthus.

Trait (units)	Description
BaseDiameter (mm)^*a*^	Ground level diameter of widest part of plant base
TransectCount (count)^*a*^	Number of stems across the middle of the plant reaching ≥50% of the canopy height
StemDiameter (mm)^*a*^	Diameter at 10–15 cm from the base of a randomly selected stem
TallestStem (cm)^*a*^	Length of the tallest stem, from the base to the uppermost ligule
MaxCanopyHght (cm)^*a*^	Maximum of canopy height values over the growing season (see below)
DOYFS1 (Julian day)^*a*^	Day of year when the first flag leaf emerged
AvgeSen (numeric score, range=0–10)^*a*^	Average score of senescence over the growing season
Moisture (%)^*a*^	Moisture content based on the weight difference between wet and dry subsamples
DryMatter (g)^*a*^	Dry biomass yield for the whole plant estimated from wet and dry subsamples
ES.DOY (numeric score, range=0–5)	Emergence score (see ‘Materials and methods’) on a given day of the year (e.g. ES.119 is the emergence score on day 119)
ESstageDOY (Julian day)	Day of the year when a given emergence score was reached
CanHght.DOY (cm)	The canopy height on a given day of the year, measured from the ground to the inflection point of most of the leaves at the plant’s top

^*a*^ Detailed description of this trait and the protocol for its measurement was provided by Slavov *et al.* (2013).

We used the molecular marker data generated by Slavov *et al.* (2014) and available on the NCBI Short Read Archive (SRP062565, PRJNA293153). Briefly, single-nucleotide polymorphisms (SNPs) were detected and genotyped through restriction site associated DNA sequencing (RAD-Seq) using the *Pst*I restriction endonuclease. The 53 174 SNPs used in this study were identified by alignment to the *Sorghum bicolor* v 1.0 genome (Paterson *et al.*, 2009) and then filtered based on sequencing depth (≥3 reads per genotype) and percentage of missing data (≤20%).

### Heritability and genotypic values

For each trait in each year, a linear mixed-effects model (LMEM) of the following form was used:

$$x_{ijkl}=b_{i}+r_{j}+c_{k}+g_{l}+e_{ijkl}$$ (1)

Where *x*_*ijkl*_ was the phenotypic value of the *l*th genotype in the *k*th column of *j*th row within the *i*th block and *e*_*ijkl*_ was the residual error effect. All terms were treated as random effects, making this approach equivalent to the PRM model of spatial variability evaluated by Robbins *et al.* (2012). The analysis was performed in R (R Core Team, 2013) using the *lmer* function of the lme4 package (Bates *et al.*, 2015). To assess the need for spatial corrections, we also fitted a simplified version of Eqn 1, excluding row and column effects. Furthermore, we compared the ability of our model to account for spatial variability to that of the two-dimensional spline (2DS) model, which is believed to be highly robust based on both simulated and empirical data (Robbins *et al.* 2012). The 2DS model was fitted using the *Tps* function of the fields package in R (Nychka *et al.* 2015). Model performance in terms of removing spatial variability, while minimizing over-fitting (i.e. confounding genetic and environmental variance; Robbins *et al.*, 2012), was measured based on the resulting estimates of broad-sense heritability:

$$H^{\text{2}}=v_{\text{g}}/\left(v_{\text{g}}+v_{\text{e}}\right)$$ (2)

where *v*_g_ and *v*_e_ were the estimated genotype and error variance components, respectively. Because the model described by Eqn 1 consistently resulted in higher *H*^2^ estimates than those from a model without within-block spatial correction and the 2DS model (Supplementary Tables S1–S4 at *JXB* online), best linear unbiased predictors (BLUPs) of genotype values from that model were used for all subsequent analyses (Supplementary Dataset S1). Likelihood profile 95% confidence intervals (CI) for *v*_g_ and *v*_e_ were calculated where possible using the *confint.merMod* function within the lme4 package. In addition, 95% CI for all *H*^2^ estimates, as well as for *v*_g_ and *v*_e_ estimates in *M. sacchariflorus* (i.e. because the likelihood profile approach failed due to the small number of genotypes) were obtained based on 100 model-based parametric bootstrap samples generated using the *bootMer* function of lme4. If a trait’s data caused a warning message during LMEM analyses and/or had *v*_g_ that was not significant at α=0.05 (i.e. 95% CI included zero), it was excluded from subsequent analyses.

### Genetic correlations and multiple linear regression of biomass yield

We calculated genetic correlations (*r*_g_) using several different approaches (Slavov *et al.*, 2014). Because *r*_g_ values calculated from estimates of genetic covariance (see Eqn 6 below and Slavov *et al.*, 2014) were outside of the expected range (−1, 1) for some of the emergence traits, we present *r*_g_ approximations calculated as Pearson’s correlation coefficients of the genotypic BLUP values (Supplementary Dataset S1). To assess how the genetic correlation between emergence time and canopy height changed over the growing season, we set a reference date when the mean phenotypic emergence score for each species first became ≥2.5 (i.e. trying to maximize the variance of emergence scores, which were in the 0–5 range; Table 1). Further checks then ensured that these reference days did not coincide with frost events and their selection did not substantially influence our results.

Although several traits were moderately correlated with DryMatter (Supplementary Dataset S1; Slavov *et al.*, 2014), none appeared to be a strong predictor. We therefore used multiple linear regression (MLR) to build biometric models with greater explanatory power for this trait, as well as for early season canopy height and maximum canopy height (i.e. because canopy height was the strongest predictor of DryMatter in this and previous studies: Supplementary Dataset S1; Robson *et al.*, 2013*b*; Slavov *et al.*, 2014). Separate MLR models were built for *M. sinensis* and *M. sacchariflorus* in both 2008 and 2009. MLR was performed at the genotypic BLUP level and started with an initial list that contained all traits that were measured chronologically before the trait being modelled. The R *step* function used in the forward direction automatically performed the initial model selection process. The resulting model was then manually pruned until the final version contained only traits significant at α=0.05. The early season canopy heights we chose to model were for day 133 in 2008 and day 138 in 2009. These dates were at or after the last emergence measurements, when the plants were exhibiting a broad range of canopy heights. Traits (usually specific emergence score days) were excluded if *v*_g_ was not significant or caused warnings in the R statistical routines used prior to the MLR (see above). For analyses aimed at identifying and comparing predictors among species, flowering time (DOYFS1) was excluded from all MLR analyses because most *M. sacchariflorus* genotypes did not flower in 2008 and 2009. However, we did include this trait in model selection procedures aimed at developing selection indices for *M. sinensis* as roughly 90% of the genotypes for that species flowered in both years.

### Genome-wide prediction

For genome-wide analyses and presentation, we followed the methodology described by Slavov *et al.* (2014). Briefly, we used the R package rrBLUP (Endelman, 2011) and random ten-fold cross-validations, with 100 iterations per trait, implemented in the R-script available at github.com/ChrisDaveyCymru/ds-gs. Predictive abilities were calculated as Pearson correlation coefficients between the ‘observed’ BLUPs and the values predicted by rrBLUP. Because RAD-Seq data were only available for the 138 *M. sinensis* genotypes, all analyses described in this and the following sections were only performed for this species.

### Selection indices integrated with genome-wide prediction

Results from this and a previous study (Slavov *et al.*, 2014) indicated that genome-wide prediction for biomass yield was largely ineffective. We therefore attempted to improve predictive ability by using the genetic correlations between biomass yield and other morphological traits that are related to this highly composite trait. To achieve this, we first constructed selection indices for yield and its best predictors (i.e. based on our MLR analyses described above) and then performed genome-wide prediction on these selection indices (Mackay *et al.*, 2015).

A selection index consists of two components (Falconer, 1989; Cameron, 1997): the selection objective (SO) and the selection index (SI). The SO contains all the traits to be selected for simultaneously when selection is based on the value of the SI. Thus, the SO can be viewed as a composite trait to be selected for. The SO has the form:

$$\text{SO}=a_{\text{1}}y_{\text{1}}+a_{\text{2}}y_{\text{2}}+\ldots +a_{n}y_{n}$$ (3)

where *y*_1_ is the first trait, *y*_2_ the second and so on to final trait *y*_*n*_, whilst *a*_1_, *a*_2_, … *a*_*n*_ are the economic values associated with a unit improvement in the respective traits.

The SI contains the traits that are measured and these can be different from the traits in the SO. The calculated value of the SI for an individual is a prediction of its genetic merit for the SO. Thus, selection of individuals for breeding based on their SI scores will improve the SO and hence all the traits it contains to an extent weighted by the economic values *a*_1_, *a*_2_, … *a*_*n*_. The SI has the form:

$$\text{SI}=w_{\text{1}}x_{\text{1}}+w_{\text{2}}x_{\text{2}}+\ldots +w_{m}x_{m}$$ (4)

where the *x* terms are the traits measured on an individual (*m* traits in total), whereas *w*_1_, *w*_2_, …, *w*_*m*_ are calculated so that selection on the SI maximizes the response in the SO. The *w* values are found by solving:

Pw= Ga (5)

where **a** is a vector of the *a*_1_, *a*_2_, … *a*_*n*_ economic values in Eqn 3, **w** is a vector of the *w*_1_, *w*_2_, …, *w*_*m*_ values in Eqn 4, P is the variance–covariance matrix for the phenotypic values of the traits in the SI, and G is the (additive) genetic covariance matrix for the traits in the SI and the SO. Thus, G provides the genetic level link between the SI and SO.

Two caveats need to be acknowledged here. Firstly, in all our analyses, we used the same traits in the SO and SI, and the matrix G in Eqn 5 was therefore a variance–covariance matrix. However, G contained the total genetic variances and covariances because our design (i.e. clonal genotype replicates) did not allow their decomposition into additive and non-additive components. Secondly, the *a*_1_, *a*_2_, … *a*_*n*_ terms in the SO (Eqn 3) were all set to 0, except for that for biomass yield, which was set to 1. Thus, the SO was in practice biomass yield alone, while other traits were only included to improve the predictive ability and response to selection for yield through their genetic correlations with this trait.

For each pair of traits (subscripts 1 and 2) in the P and G matrices, LMEMs were used to estimate their respective genetic (*v*_g1_ and *v*_g2_) and residual error variances (*v*_e1_ and *v*_e2_). Using the properties of linear functions of random variables (Wackerly *et al.*, 2002), the LMEM described in Eqn 1 was then fitted on the sum of the two traits and their genetic covariance (cov_g12_) was calculated as:

$${\text{cov}}_{\text{g12}}=\left(v_{\text{gs}}–v_{\text{g1}}–\;v_{\text{g2}}\right)/\text{2}$$ (6)

where *v*_gs_ was the genetic variance of the sum of the two traits (Howe *et al.*, 2000). Residual error variances and covariances were estimated using the same approach, and then the G and P matrices were populated with the appropriate terms (i.e. with phenotypic (co-) variances expressed as the sums of the respective genetic and residual error (co-) variances).

After solving Eqn 5 for the *w*_1_, *w*_2_, …, *w*_*m*_ terms, the SI value for each individual plant was calculated using Eqn 4, and the resulting SI values were treated as phenotypic traits to calculate *H*^2^, extract genotypic BLUPs and perform genome-wide prediction (see above). Following Falconer (1989), the relative response to selection *R*_rel_ was then calculated as:

$$R_{\text{rel}}=\sqrt{v_{\text{SI}}}/(\sqrt{H^{2}}\;\sqrt{v_{\text{g}}})$$ (7)

where *H*^2^ and *v*_g_ were the broad-sense heritability and genetic variance for dry matter yield, whereas *v*_SI_ was the variance of the SI, which was calculated as:

$$v_{\text{SI}}=w_{\text{1}}v_{\text{g1}}+w_{\text{2}}{\text{cov}}_{\text{g12}}+w_{\text{3}}{\text{cov}}_{\text{g13}}\ldots +w_{m}{\text{cov}}_{\text{g1}m}$$ (8)

Trait 1 was always dry matter yield, and all covariance terms were therefore between the additional traits in the SI and yield.

A schematic diagram of the custom R-scripts used to perform all analyses in this section is shown in Supplementary Fig. S1 and the R-script used to calculate the selection indices is available at github.com/ChrisDaveyCymru/ds-si. The traits considered for the SIs were identified by our MLR models (see above), although we also considered more parsimonious (and therefore less demanding in terms of additional phenotyping) SIs, including only core subsets of the significant yield predictors.

## Results and discussion

### Genetic determination and relationship between emergence, canopy height and yield

**Fig. 1.**
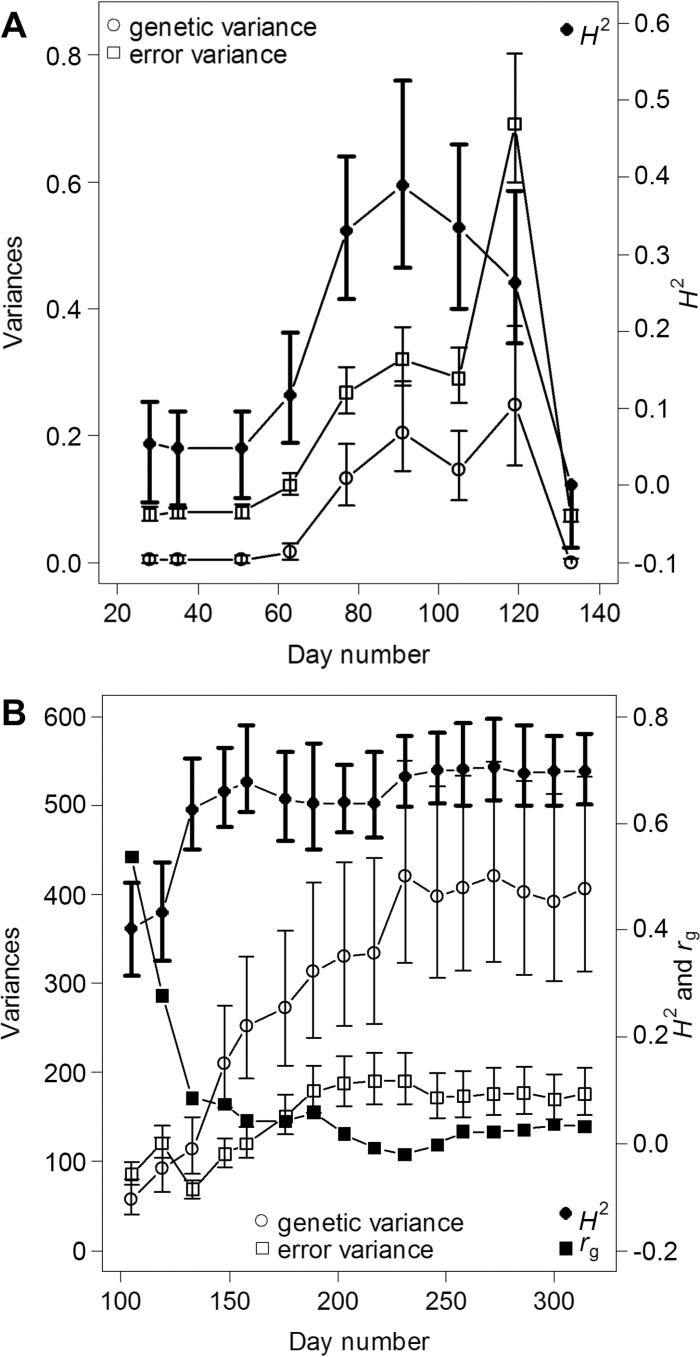
Variance components and broad sense heritabilities (*H*^2^) for emergence score (A) and canopy height (B) *versus* day in the year for *M. sinensis* in 2008, and genetic correlation (*r*_g_) of canopy height with emergence score on day 91 (B). The peak in error variance on day 119 in (A) was due to a frost event. Error bars correspond to 95% confidence intervals (see ‘Materials and methods’).

Most of the phenotypic traits that we studied had a moderate to strong degree of genetic determination (Table 2 and Supplementary Tables S1–S4). As expected, broad sense heritabilities (*H*^2^) for biomass-related morphological traits (i.e. BaseDiameter, TransectCount, StemDiameter and MaxCanopyHght) increased slightly as the crop matured from 2008 to 2009, but this was not the case for biomass yield (DryMatter). Heritabilities for emergence score were time dependent, with a clear peak that tended to be in the range 0.51–0.54 for both *M. sinensis* and *M. sacchariflorus* (Supplementary Tables S1–S4), although the value for *M. sinensis* in 2008 was slightly smaller at 0.39 (Fig. 1A, Supplementary Table S1). The peaks occurred between days 82 and 96 for both species across both years and corresponded to emergence score 3–4 (shoot elongation to leaf emergence) in *M. sinensis* and score 1 (initial bud/shoot emergence from the soil) in *M. sacchariflorus*. In contrast, the *H*^2^ values for canopy height rapidly increased to a plateau value (Fig. 1B). For *M. sinensis* this plateau value was about 0.70 in both years (Fig. 1B, Supplementary Tables S1 and S2), while for *M. sacchariflorus* the value was substantially higher at 0.91–0.93 (Supplementary Tables S3 and S4). Thus, the relative degree of genetic determination of both emergence and canopy height is dynamic, and care should be taken when selecting dates for measuring these traits. It is encouraging, however, that the optimal time for capturing emergence data was consistently within a relatively narrow 2-week window as this allows phenotyping activities to be planned with precision.

**Table 2.** *Broad sense heritabilities* (H^*2*^*) for 138* M. sinensis *(sin) and 30* M. sacchariflorus *(sac) genotypes in 2008 (.08) and 2009 (.09*). Traits are described in Table 1. Heritabilities for emergence score and canopy heights over time, as well as variance component estimates and comparisons of different spatial correction models, are shown in Supplementary Tables S1–S4. Model-based parametric bootstrap 95% confidence intervals (95% CI) are shown in brackets (see ‘Materials and methods’).

Trait name	*H* ^2^ sin.08 (95% CI)	*H* ^2^ sin.09 (95% CI)	*H* ^2^ sac.08 (95% CI)	*H* ^2^ sac.09 (95% CI)
BaseDiameter	0.358 (0.270, 0.480)	0.522 (0.431, 0.616)	0.330 (0.109, 0.534)	0.451 (0.242, 0.732)
TransectCount	0.490 (0.380, 0.581)	0.528 (0.453, 0.622)	0.556 (0.367, 0.773)	0.746 (0.646, 0.940)
StemDiameter	0.477 (0.380, 0.573)	0.619 (0.547, 0.698)	0.710 (0.601, 0.857)	0.885 (0.835, 0.980)
TallestStem	0.849 (0.816, 0.886)	0.883 (0.856, 0.915)	0.852 (0.794, 0.959)	0.704 (0.582, 0.907)
MaxCanopyHght	0.685 (0.630, 0.747)	0.799 (0.760, 0.862)	0.909 (0.860, 1.004)	0.924 (0.889, 0.991)
DOYFS1	0.943 (0.928, 0.960)	0.890 (0.858, 0.926)	0.749 (0.498, 1.155)^*a*^	0.959 (0.917, 1.058)
AvgeSen	0.901 (0.878, 0.926)	0.832 (0.794, 0.887)	0.916 (0.876, 0.965)	0.884 (0.830, 0.968)
Moisture	0.871 (0.833, 0.904)	0.805 (0.761, 0.855)	0.205 (0.000, 0.411)^*a*^	0.619 (0.461, 0.884)
DryMatter	0.587 (0.519, 0.674)	0.571 (0.482, 0.653)	0.679 (0.532, 0.868)	0.680 (0.531, 0.869)
ES1DOY	0.042 (-0.038, 0.084)^*a*^	0.228 (0.133, 0.319)	0.606 (0.442, 0.794)	0.432 (0.274, 0.635)
ES2DOY	0.042 (-0.028, 0.084)^*a*^	0.228 (0.134, 0.324)	0.460 (0.261, 0.695)	0.230 (0.016, 0.400)
ES3DOY	0.478 (0.392, 0.569)	0.416 (0.331, 0.517)	0.450 (0.223, 0.683)	0.235 (0.014, 0.464)
ES4DOY	0.373 (0.288, 0.465)	0.521 (0.449, 0.619)	0.443 (0.261, 0.732)	0.432 (0.253, 0.690)
ES5DOY	0.359 (0.263, 0.475)	0.404 (0.327, 0.500)	0.446 (0.265, 0.679)	0.005 (-0.188, 0.011)^*a*^

^*a*^ Genetic variance not significant or generated an R warning when used in a relevant statistical function.

To characterize the variability of genetic correlations between emergence and canopy heights during the growing season, the emergence score on a reference day early in the growing season was selected as described in ‘Materials and methods’. For both *M. sinensis* and *M. sacchariflorus* in 2008 the reference day was day 91 (31 March). In 2009, the reference day for *M. sinensis* was 82 (23 March) and for *M. sacchariflorus* 96 (6 April), reflecting the differing weather, growth and measurement days in the two years. Early-season canopy heights were moderately correlated with emergence, but these correlations rapidly declined in both 2008 (Fig. 1B) and 2009 (Supplementary Dataset S1). This suggested that early-season growth rate may be a more important determinant of canopy height, and ultimately biomass yield, than the starting date *per se* (Supplementary Dataset S1). Consistent with this result, the importance of the early-season growth rate for yield production in *Miscanthus* was also suggested by Farrell *et al.* (2006).

The weak genetic association between emergence and yield (Supplementary Dataset S1), presumably mediated by the transient genetic correlation of emergence with canopy height, indicated that yield could not be reliably predicted solely based on spring phenology. We therefore used MLR models to identify the key predictors of biomass yield and clarify the relative importance of spring events (Table 3, Supplementary Table S5). Emergence was an important predictor of early-season canopy height in both *M. sinensis* and *M. sacchariflorus*. In addition, plant basal diameter was a key predictor for the former species and stem diameter for the latter. As expected, maximum canopy height was more complex, with emergence being an important predictor in *M. sinensis* and to a lesser extent in *M. sacchariflorus*, while stem diameter was a significant predictor in both species. Finally, the MLR models of dry biomass yield indicated that canopy height (both early and later in the growing season) and stem number (i.e. TransectCount) were by far the most robust predictors. In *M. sinensis*, senescence and to a lesser extent emergence were also significant predictors, whereas neither was significant in *M. sacchariflorus*. Overall, our MLR models confirmed indications from the genetic correlation analyses that (i) the timing of emergence was a significant predictor of early-season elongation growth, but less so for growth later in the season and for the final dry biomass yield; (ii) early-season canopy height was consistently more informative than emergence for predicting biomass yield; and (iii) there were substantial species differences with respect to the relative importance of phenological and morphometric traits as determinants of biomass yield.

**Table 3.** *Generalized summary of the multiple linear regression models for early season canopy heights (CanHght.133 in 2008 and CanHght.138 in 2009), max canopy heights (MaxCanopyHght) and dry matter yield (DryMatter) for* M. sinensis *(sin) and* M. sacchariflorus *(sac) in 2008 (08) and 2009 (09*). The traits in the final models have been grouped under over-arching headings to emphasize the trends. The actual traits in the models are listed in Supplementary Table S5.

Trait	Species (year)	Adjusted *R*^2^	Emergence	Senescence (moisture)	Base diameter of plant	Transect count	Stem diameter	Canopy height at ≤180 day of year	Canopy height at >180 day of year	Tallest stem, max canopy height
Early canopy height	Sin (08)	0.39	✓		✓	✓				
	Sin (09)	0.30	✓		✓					
	Sac (08)	0.76	✓				✓			
	Sac (09)	0.64	✓				✓			
Max canopy height	Sin (08)	0.34	✓				✓			
	Sin (09)	0.44	✓	✓	✓		✓			
	Sac (08)	0.90	✓				✓			
	Sac (09)	0.87					✓			
Dry matter yield	Sin (08)	0.76		✓	✓	✓		✓	✓	
	Sin (09)	0.71	✓	✓		✓	✓	✓	✓	✓
	Sac (08)	0.86				✓		✓	✓	
	Sac (09)	0.79				✓		✓	✓	✓

### Genome-wide prediction

Thus far it has been established that canopy phenology and, to a lesser extent, emergence are important determinants of biomass yield and that all of these traits are heritable. Our next step was to build on this by exploiting a genome-wide SNP set for the *M. sinensis* genotypes used here in order to study these traits at the genomic level. Firstly, exploratory genome-wide association study (GWAS) analyses identified a large number of SNPs, with relatively small individual effects, associated with emergence after Bonferroni adjustments (data not shown), but these results will be reported separately, after re-analyses in larger GWAS populations (*n*=1000 genotypes).

**Fig. 2.**
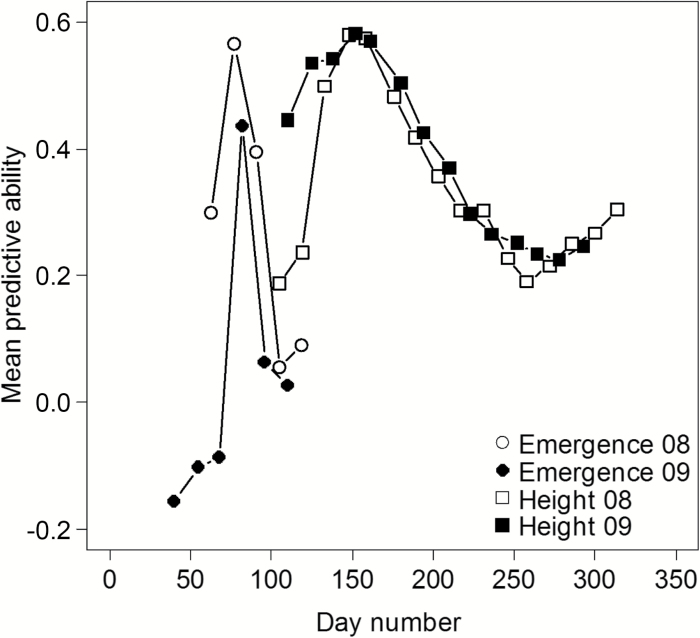
Genome-wide prediction of emergence score and canopy height versus day of year for M. sinensis in 2008 and 2009

Secondly, we performed genome-wide prediction to explore the practical value of our research to plant breeders. As expected from our previous study (Slavov *et al.*, 2014), genome-wide predictive abilities based on random cross-validations ranged from moderate to high for most traits (Table 4), reaching as high as 0.76 for the traits Moisture and DOYFS1 (see Table 1). However, the predictive ability for dry biomass yield was close to zero. This was not surprising since yield depends so strongly on the cumulative interactions of many sub-traits with the environment over the whole growing season. Also, the plants were harvested in the late winter, which means that the yield values also reflected the stochastic losses of stem material in winter storms. For emergence score and canopy height, data were available through multiple time points. For these traits, it was therefore possible to look at the time dependence of predictive ability, which in both cases had peaks that were highly repeatable across the two years (Fig. 2). As expected, the peak of predictive ability for emergence score roughly corresponded to the peak in *H*^2^. In 2008 the peak predictive ability for canopy height was on day 148 and in 2009 on day 152, but in both cases the equivalent median canopy height was 65 cm. Thus, we have identified both the optimal time and developmental stage for collecting spring phenology data under the conditions of our field trial, thereby substantially reducing the time and cost of future measurements.

**Table 4.** *Genome-wide predictive abilities for* M. sinensis *in 2008 and 2009*.

Trait	Mean (SD) predictive ability	
	2008	2009
BaseDiameter	0.30 (0.04)	0.28 (0.05)
TransectCount	0.27 (0.03)	0.16 (0.04)
StemDiameter	0.43 (0.04)	0.51 (0.03)
TallestStem	0.64 (0.01)	0.65 (0.01)
MaxCanopyHght	0.16 (0.04)	0.37 (0.02)
DOYFS1	0.73 (0.02)	0.76 (0.02)
AvgeSen	0.64 (0.01)	0.64 (0.01)
Moisture	0.74 (0.01)	0.76 (0.01)
DryMatter	0.01 (0.05)	0.06 (0.05)
ES1DOY	—	−0.13 (0.10)
ES2DOY	—	−0.13 (0.10)
ES3DOY	0.49 (0.02)	−0.08 (0.02)
ES4DOY	0.43 (0.03)	0.41 (0.03)
ES5DOY	0.25 (0.05)	0.32 (0.06)

### Selection indices integrated with genome-wide prediction

**Fig. 3.**
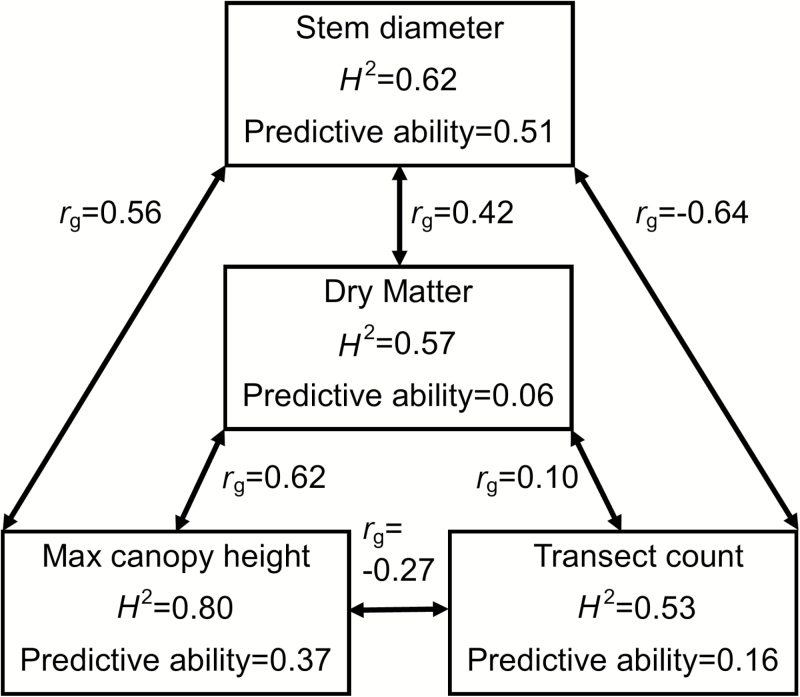
Conceptual model of the ‘Total stem volume’ selection index for dry matter yield using the data for *M. sinensis* in 2009 (Table 5, index 2d). The yield of a *Miscanthus* plant is approximated as the total stem volume using the three additional traits in the figure.

One general approach to improving genome-wide predictions of biomass yield is to exploit its genetic correlations with other traits that have higher *H*^2^ and/or predictive abilities (Fig. 3). More specifically, we implemented a variation of the idea suggested by Mackay *et al.* (2015), by first building selection indices (SIs), with biomass yield being the only selection objective (see ‘Materials and methods’), and then performing genome-wide prediction on these SIs. Using the relative response to selection (i.e. compared with direct selection on yield) and genome-wide predictive ability of each SI as benchmarks, we evaluated several different scenarios, using the key predictor traits for yield identified by our MLR models (see above, Table 3 and Supplementary Table S5) and exploring scenarios aimed at minimizing phenotyping effort (Table 5).

**Table 5.** *Component traits, broad-sense heritabilities* (H^*2*^*), relative responses* (R_*rel*_*) and genome-wide predictive abilities of selection indices aimed at increasing gains of biomass yield (DryMatter) of* M. sinensis *in 2008 and 2009*.

Type/name of index	Traits included in addition to DryMatter	Year	*R*_rel_	*H* ^2^	Predictive ability (SD)
**1.Phenological**					
1a. Emergence	ES4DOY	2008	1.00	0.59	0.04 (0.06)
	ES4DOY	2009	1.02	0.56	0.07 (0.05)
1b. Early growth	CanHght.158	2008	1.00	0.59	0.04 (0.06)
	CanHght.161	2009	1.00	0.57	0.07 (0.06)
1c. Flowering	DOYFS1	2008	1.15	0.74	0.41 (0.02)
	DOYFS1	2009	1.06	0.66	0.32 (0.03)
1d. Senescence	AvgeSen	2008	1.03	0.67	0.34 (0.04)
	AvgeSen	2009	1.01	0.60	0.21 (0.05)
1e. Canopy duration	ES4DOY, AvgeSen	2008	1.03	0.67	0.34 (0.04)
	ES4DOY, AvgeSen	2009	1.03	0.59	0.19 (0.05)
**2.Morphometrical**					
2a. Stem diameter	StemDiameter	2008	0.89	0.53	−0.10 (0.06)
	StemDiameter	2009	1.02	0.62	0.23 (0.05)
2b. Stem number	TransectCount	2008	1.01	0.62	0.17 (0.05)
	TransectCount	2009	1.01	0.60	0.21 (0.05)
2c. Total stem diameter	TransectCount, StemDiameter	2008	0.98	0.59	0.13 (0.05)
	TransectCount, StemDiameter	2009	1.03	0.63	0.25 (0.05)
2d. Total stem volume	TransectCount, StemDiameter, MaxCanopyHght	2008	1.00	0.60	0.18 (0.05)
	TransectCount, StemDiameter, MaxCanopyHght	2009	1.03	0.64	0.25 (0.05)
2e. Plant volume	StemDiameter, TallestStem, BaseDiameter	2008	0.97	0.57	0.13 (0.05)
	StemDiameter, TallestStem, BaseDiameter	2009	0.89	0.48	0.36 (0.02)
**3.Combined (MLR**)					
3a. Reduced	DOYFS1, TransectCount	2008	1.16	0.75	0.42 (0.02)
	DOYFS1, TransectCount	2009	1.07	0.68	0.36 (0.03)
3b. Full	ES4DOY, CanHght.176, DOYFS1, AvgeSen, TransectCount, StemDiameter, TallestStem, BaseDiameter	2008	1.21	0.79	0.45 (0.02)
	ES4DOY, CanHght.180, DOYFS1, AvgeSen, TransectCount, StemDiameter, TallestStem, BaseDiameter	2009	1.16	0.69	0.36 (0.03)

Several generalizations emerged from these analyses. Firstly, as expected from our correlation and MLR analyses, SIs based exclusively on spring phenology traits (i.e. emergence and early elongation growth) did not result in practically significant improvements in the relative response to selection or genome-wide predictive ability (Table 5, indices 1a and 1b). In contrast, SIs that included both spring and fall events had consistently higher relative responses to selection (i.e. by 1–15%) and increased genome-wide predictive ability by an order of magnitude (Table 5, indices 1c–1e). Most notably, a very parsimonious SI, including flowering time alone (Table 5, index 1c), was nearly as effective as the best SI we identified using MLR (Table 5, index 3b). Thus, although measurements of flowering time require multiple field surveys over extended periods of time (Jensen *et al.*, 2011), capturing even a crude measure of flowering phenology using a high-throughput phenotyping platform may enable the implementation of this SI. Alternatively, SIs including measures of both emergence and senescence (i.e. canopy duration, Table 5, index 1e) may be feasible, as these traits could be captured in single, well-chosen time points (see above) or via automated imaging. Secondly, SIs based exclusively on morphometrical traits (Table 5, indices 2a–2e) performed less consistently and generally worse than phenological SIs. Interestingly, the genome-wide predictive abilities for all five morphometrical SIs improved considerably (i.e. by ~1–5 standard deviations) from 2008 to 2009, suggesting that these SIs may be more effective once crop maturity is reached. Finally, a ‘full model’ SI, which included both phenological and morphometrical traits that were identified as significant predictors of yield in our MLR analyses, resulted in 16% higher relative response to selection and six-fold increase in genome-wide predictive ability in 2009, with even better performance in 2008 (Table 5, index 3b). The considerable phenotyping effort required for this SI will probably make it impractical in most situations, although this may not be the case if the use of automated high-throughput phenotyping platforms becomes common in breeding programmes. More importantly, a ‘reduced model’ SI, which included only flowering time and stem number, resulted in a similar level of improvement (Table 5, index 3a), suggesting that simplified SIs (e.g. Table 5 indices 1c–1e, 2b and 3a) could readily be implemented in most breeding programmes.

### Implications

The findings of this study, and particularly the results of combining SIs and genome-wide prediction, have several important practical implications for breeding *Miscanthus* and other perennial biomass crops. Firstly, the length of time needed for the plants to mature enough to evaluate their merit for selection as parents is probably the biggest limiting factor for accelerating the domestication of *Miscanthus.* Genomic selection is therefore likely to play a particularly important role in shortening breeding cycles. Although genome-wide prediction of biomass yield was ineffective in *M. sinensis* (Slavov *et al.*, 2014), the addition of even a single phenotypic trait (e.g. flowering time, senescence or stem number) in our simplest SI scenarios led to several-fold increases in predictive ability, with the added benefit of slightly increased response to selection (Table 5). Thus, the challenge of using genomic selection to increase biomass yield can be overcome with very modest additional phenotyping effort (i.e. through a single field survey). It is currently unclear, however, if it is feasible to implement this approach in ongoing breeding programmes. This is because genome-wide prediction has not yet been evaluated across multiple generations and in inter-specific hybrids (i.e. the most likely market products). Both of these uncertainties are being assessed in an ongoing study using larger *Miscanthus* populations (*n*=1000 genotypes), including all widely distributed species and a set of highly productive inter-specific hybrids. Secondly, early breeding efforts in *Miscanthus* have focused exclusively on increasing biomass yield, but other traits, such as seed-based propagation to reduce establishment cost (or its absence to reduce invasiveness), abiotic stress resistance and biomass composition are likely to become increasingly important in the future (Clifton-Brown *et al.*, 2017). For example, selection indices have already been used to simultaneously improve biomass yield and quality in switchgrass (Jahufer and Casler, 2015). Thus, our approach of combining genome-wide prediction and SIs would be a natural choice for accelerated breeding programmes that target multiple traits. Finally, at a more fundamental level, the integration of SIs and genomic selection can also be used to predict key parameters used in mathematical models of plant growth and yield (e.g. Davey *et al.*, 2016), and so provide a direct link between genetics and physiological process-based modelling (Yin and Struik, 2016). Recent studies have demonstrated the potential of integrating crop growth models with genomic selection, particularly for prediction in the presence of strong genotype-by-environment interactions (Technow *et al.*, 2015; Cooper *et al.*, 2016; Messina *et al.*, 2017). The critical factor for the accuracy of these models, however, appears to be the availability of training data sets from multiple contrasting environments (Messina *et al.*, 2017). Our SI-based approach could readily be applied to predict the physiological parameters underlying crop growth models from more easily measurable phenotypic traits (i.e. potentially including traits captured through high-throughut phenotyping platforms). It could therefore play an important role in the development of a highly integrated analytical framework, which would enable large-scale virtual breeding and testing experiments, thereby transforming the efficiency of plant breeding (Messina *et al.*, 2017).

## Supplementary data

Supplementary data are available at *JXB* online.

Dataset S1. Heat maps of the phenotypic and genetic correlations for the traits in Table 1 as well as tables of the trait BLUPs for *M. sinensis* and *M. sacchariflorus* in 2008 and 2009.

Fig. S1. Flowchart of the data analysis for the selection index calculations and subsequent genome-wide prediction.

Table S1. Broad sense heritabilities of all traits measured in *M. sinensis* during 2008.

Table S2. Broad sense heritabilities of all traits measured in *M. sinensis* during 2009.

Table S3. Broad sense heritabilities of all traits measured in *M. sacchariflorus* during 2008.

Table S4. Broad sense heritabilities of all traits measured in *M. sacchariflorus* during 2009.

Table S5. Individual traits selected in the multiple linear regression models shown as generalized summaries in Table 3.

## Supplementary Material

Supplementary Data

Supplementary Tables and Figure
