# Supplementary material for: Genetic relationships between spring emergence, canopy phenology, and biomass yield increase the accuracy of genomic prediction in Miscanthus
Source: J Exp Bot. 2017 Oct 12;68(18):5093–102. doi: 10.1093/jxb/erx339 (PMC5853932; doi:10.1093/jxb/erx339)
Supplement: Supplementary Tables and Figure [file erx339_suppl_supplementary_tables_s1_s5_figure_s1.pdf]

Table S1. Broad sense heritabilities of all traits measured in *M. sinensis* during 2008 based on mixed models with spatial corrections based on (1) a two-dimensional spline (H^2_{2DS}), (2) random block effects only (H^2_B) and (3) random block, row and column effects (H^2_{BRC}). The latter model (values in bold) was chosen for all subsequent analyses, and estimates of genetic (v_g) and residual error (v_e) variance, as well as genotypic best linear unbiased predictors (BLUPs), were obtained from that model. Likelihood profile or model-based parametric bootstrap 95% confidence intervals (95% CI) are shown in brackets (see Materials and methods).

Trait	H^2_{2DS}	H^2_B	**H^2_{BRC} [95% CI]**	v_g [95% CI]	v_e [95% CI]
BaseDiameter	0.254	0.331	**0.358 [0.270, 0.480]**	2174.98 [1497.18, 3084.36]	3906.75 [3389.59, 4534.35]
TransectCount	0.152	0.467	**0.490 [0.380, 0.581]**	31.95 [23.51, 43.43]	33.30 [28.86, 38.61]
TallestStem	0.819	0.839	**0.849 [0.816, 0.886]**	1434.00 [1123.59, 1845.32]	254.48 [219.92, 296.71]
MaxCanopyHght	0.631	0.667	**0.685 [0.630, 0.747]**	453.09 [348.25, 593.49]	208.14 [180.31, 242.07]
StemDiameter	0.472	0.476	**0.477 [0.380, 0.573]**	0.87 [0.64, 1.18]	0.96 [0.83, 1.11]
Moisture	0.859	0.864	**0.871 [0.833, 0.904]**	50.23 [39.31, 64.49]	7.45 [6.44, 8.68]
DryMatter	0.494	0.561	**0.587 [0.519, 0.674]**	129248.33 [97268.32, 172565.98]	90761.13 [78502.58, 105729.76]
DOYFS1	0.936	0.939	**0.943 [0.928, 0.960]**	992.31 [768.83, 1286.03]	59.57 [50.81, 70.45]
AvgeSen	0.876	0.896	**0.901 [0.878, 0.926]**	0.99 [0.78, 1.27]	0.11 [0.09, 0.13]
ES.28	0.042	0.042	**0.054 [-0.023, 0.108]***	0.00 [0.00, 0.01]*	0.08 [0.07, 0.09]
ES.35	0.037	0.038	**0.048 [-0.030, 0.096]***	0.00 [0.00, 0.01]*	0.08 [0.07, 0.09]
ES.51	0.037	0.038	**0.048 [-0.017, 0.096]***	0.00 [0.00, 0.01]*	0.08 [0.07, 0.09]
ES.63	0.106	0.112	**0.117 [0.055, 0.198]**	0.02 [0.00, 0.03]	0.12 [0.11, 0.14]
ES.77	0.322	0.330	**0.330 [0.242, 0.427]**	0.13 [0.09, 0.19]	0.27 [0.23, 0.31]
ES.91	0.344	0.363	**0.390 [0.283, 0.525]**	0.20 [0.14, 0.28]	0.32 [0.28, 0.37]
ES.105	0.328	0.328	**0.335 [0.229, 0.443]**	0.15 [0.10, 0.21]	0.29 [0.25, 0.34]
ES.119	0.119	0.193	**0.264 [0.184, 0.383]**	0.25 [0.15, 0.37]	0.69 [0.60, 0.80]
ES.133	0.000	0.000	**0.000 [-0.081, 0.000]***	0.00 [0.00, 0.01]*	0.07 [0.06, 0.08]
ES1DOY	0.032	0.032	**0.042 [-0.038, 0.084]***	4.62 [0.00, 14.12]*	105.44 [91.78, 122.23]
ES2DOY	0.032	0.032	**0.042 [-0.028, 0.084]***	4.62 [0.00, 14.12]*	105.44 [91.78, 122.23]
ES3DOY	0.465	0.476	**0.478 [0.392, 0.569]**	72.59 [53.18, 98.09]	79.23 [68.82, 91.74]
ES4DOY	0.366	0.372	**0.373 [0.288, 0.465]**	20.11 [14.02, 28.00]	33.77 [29.37, 39.12]
ES5DOY	0.347	0.350	**0.359 [0.263, 0.475]**	16.92 [11.65, 23.71]	30.19 [26.19, 35.13]
CanHght.105	0.385	0.396	**0.403 [0.314, 0.489]**	57.68 [40.88, 79.69]	85.53 [74.17, 99.4]
CanHght.119	0.422	0.431	**0.434 [0.342, 0.527]**	92.10 [65.85, 126.60]	120.30 [104.32, 139.75]
CanHght.133	0.598	0.608	**0.625 [0.551, 0.721]**	113.43 [86.02, 149.47]	68.16 [59.01, 79.36]
CanHght.148	0.626	0.642	**0.660 [0.594, 0.741]**	209.90 [160.49, 275.42]	108.23 [93.70, 125.98]
CanHght.158	0.638	0.657	**0.678 [0.621, 0.784]**	252.33 [193.51, 330.50]	119.85 [103.75, 139.52]
CanHght.176	0.599	0.626	**0.644 [0.567, 0.734]**	272.50 [207.90, 358.80]	150.36 [130.23, 174.88]
CanHght.189	0.575	0.616	**0.637 [0.552, 0.750]**	313.36 [238.85, 413.45]	178.88 [154.85, 208.16]
CanHght.203	0.582	0.617	**0.638 [0.583, 0.709]**	330.71 [251.99, 436.22]	187.32 [162.10, 218.11]
CanHght.217	0.573	0.619	**0.637 [0.572, 0.733]**	333.95 [254.55, 440.74]	190.48 [164.84, 221.78]
CanHght.231	0.630	0.665	**0.688 [0.631, 0.764]**	420.18 [322.95, 550.49]	190.36 [164.74, 221.68]
CanHght.246	0.601	0.666	**0.699 [0.637, 0.770]**	397.74 [306.35, 521.17]	171.56 [148.47, 199.74]
CanHght.258	0.637	0.679	**0.701 [0.633, 0.787]**	407.80 [314.24, 533.51]	173.71 [150.41, 202.14]
CanHght.272	0.648	0.683	**0.705 [0.643, 0.795]**	420.43 [323.98, 549.66]	175.99 [152.41, 204.74]
CanHght.286	0.613	0.671	**0.694 [0.633, 0.783]**	402.91 [310.14, 528.02]	177.35 [153.62, 206.27]
CanHght.300	0.605	0.672	**0.697 [0.633, 0.763]**	392.04 [302.06, 513.57]	170.08 [147.29, 197.83]
CanHght.314	0.630	0.671	**0.698 [0.636, 0.768]**	406.68 [313.16, 532.33]	176.06 [152.50, 204.79]
**Average (SD)**	**0.447 (0.259)**	**0.479 (0.261)**	**0.494 (0.263)**		

* v_g not significant or generated an R warning when used in a relevant statistical function.

Table S2. Broad sense heritabilities of all traits measured in *M. sinensis* during 2009 based on mixed models with spatial corrections based on (1) a two-dimensional spline (H^2_{2DS}), (2) random block effects only (H^2_B) and (3) random block, row and column effects (H^2_{BRC}). The latter model (values in bold) was chosen for all subsequent analyses, and estimates of genetic (v_g) and residual error (v_e) variance, as well as genotypic best linear unbiased predictors (BLUPs), were obtained from that model. Likelihood profile or model-based parametric bootstrap 95% confidence intervals (95% CI) are shown in brackets (see Materials and methods).

Trait	H^2_{2DS}	H^2_B	**H^2_{BRC} [95% CI]**	v_g [95% CI]	v_e [95% CI]
DOYFS1	0.885	0.889	**0.890 [0.858, 0.926]**	861.35 [666.27, 1117.69]	106.36 [90.96, 124.98]
AvgeSen	0.828	0.828	**0.832 [0.794, 0.887]**	1.18 [0.92, 1.51]	0.24 [0.21, 0.28]
BaseDiameter	0.509	0.515	**0.522 [0.431, 0.616]**	3201.20 [2363.28, 4302.23]	2929.40 [2541.10, 3403.81]
DryMatter	0.463	0.540	**0.571 [0.482, 0.653]**	158244.96 [118564.57, 212006.21]	119127.55 [102943.79, 138915.32]
MaxCanopyHght	0.706	0.774	**0.799 [0.760, 0.862]**	461.73 [360.35, 598.54]	115.99 [100.29, 135.18]
Moisture	0.559	0.590	**0.805 [0.761, 0.855]**	35.42 [27.60, 45.97]	8.56 [7.39, 9.99]
StemDiameter	0.582	0.603	**0.619 [0.547, 0.698]**	0.84 [0.64, 1.11]	0.52 [0.45, 0.60]
TallestStem	0.857	0.877	**0.883 [0.856, 0.915]**	1596.91 [1255.02, 2049.27]	211.84 [183.15, 246.87]
TransectCount	0.483	0.505	**0.528 [0.453, 0.622]**	40.96 [30.40, 55.03]	36.58 [31.67, 42.57]
ES.40	0.174	0.174	**0.191 [0.102, 0.290]**	0.03 [0.02, 0.05]	0.13 [0.11, 0.15]
ES.55	0.250	0.253	**0.259 [0.164, 0.371]**	0.03 [0.02, 0.05]	0.10 [0.09, 0.11]
ES.68	0.143	0.145	**0.154 [0.070, 0.223]**	0.02 [0.01, 0.04]	0.12 [0.10, 0.14]
ES.82	0.541	0.542	**0.545 [0.479, 0.624]**	0.27 [0.20, 0.35]	0.22 [0.19, 0.26]
ES.96	0.328	0.338	**0.357 [0.284, 0.439]**	0.13 [0.09, 0.18]	0.23 [0.20, 0.27]
ES.110	0.263	0.264	**0.264 [0.176, 0.372]**	0.02 [0.01, 0.03]	0.06 [0.06, 0.07]
ES1DOY	0.218	0.219	**0.228 [0.133, 0.319]**	12.72 [7.05, 19.79]	42.95 [37.20, 50.14]
ES2DOY	0.218	0.219	**0.228 [0.134, 0.324]**	12.72 [7.05, 19.79]	42.95 [37.20, 50.14]
ES3DOY	0.410	0.412	**0.416 [0.331, 0.517]**	8.30 [5.91, 11.37]	11.63 [10.13, 13.48]
ES4DOY	0.519	0.519	**0.521 [0.449, 0.619]**	32.82 [24.26, 43.79]	30.21 [26.33, 34.91]
ES5DOY	0.360	0.379	**0.404 [0.327, 0.500]**	23.69 [16.60, 33.07]	34.91 [30.16, 40.74]
CanHght.110	0.349	0.356	**0.370 [0.269, 0.463]**	68.36 [47.41, 95.82]	116.47 [101.08, 135.32]
CanHght.125	0.371	0.441	**0.472 [0.379, 0.584]**	62.12 [45.41, 84.67]	69.62 [60.32, 80.94]
CanHght.138	0.464	0.508	**0.530 [0.447, 0.638]**	101.47 [75.58, 136.29]	89.80 [77.82, 104.38]
CanHght.152	0.511	0.563	**0.581 [0.499, 0.656]**	253.58 [191.34, 337.50]	183.20 [158.83, 212.82]
CanHght.161	0.561	0.612	**0.623 [0.556, 0.728]**	327.69 [249.58, 432.88]	197.96 [171.55, 230.10]
CanHght.180	0.595	0.664	**0.677 [0.615, 0.728]**	456.58 [350.97, 599.22]	217.86 [188.66, 253.40]
CanHght.194	0.581	0.632	**0.653 [0.585, 0.741]**	424.18 [324.64, 558.22]	225.08 [194.95, 261.80]
CanHght.210	0.601	0.667	**0.684 [0.621, 0.756]**	457.81 [352.34, 600.20]	211.29 [183.08, 245.64]
CanHght.223	0.623	0.683	**0.693 [0.627, 0.781]**	460.62 [355.13, 602.82]	203.73 [176.50, 236.89]
CanHght.236	0.643	0.689	**0.705 [0.653, 0.786]**	493.80 [381.13, 645.14]	206.34 [178.72, 239.99]
CanHght.252	0.625	0.685	**0.698 [0.629, 0.762]**	493.78 [380.85, 646.01]	214.13 [185.45, 249.09]
CanHght.264	0.648	0.688	**0.700 [0.631, 0.796]**	491.69 [379.09, 642.65]	211.07 [182.82, 245.53]
CanHght.278	0.656	0.696	**0.704 [0.645, 0.772]**	496.28 [382.96, 648.17]	208.80 [180.81, 242.93]
CanHght.293	0.654	0.692	**0.702 [0.642, 0.775]**	512.16 [394.98, 669.09]	217.65 [188.56, 253.07]
**Average (SD)**	**0.505 (0.188)**	**0.534 (0.199)**	**0.553 (0.203)**		

* v_g not significant or generated an R warning when used in a relevant statistical function.

Table S3. Broad sense heritabilities of all traits measured in *M. sacchariflorus* during 2008 based on mixed models with spatial corrections based on (1) a two-dimensional spline (H^2_{2DS}), (2) random block effects only (H^2_{B}) and (3) random block, row and column effects (H^2_{BRC}). The latter model (values in bold) was chosen for all subsequent analyses, and estimates of genetic (v_g) and residual error (v_e) variance, as well as genotypic best linear unbiased predictors (BLUPs), were obtained from that model. Model-based parametric bootstrap 95% confidence intervals (95% CI) are shown in brackets (see Materials and methods).

Trait	H^2_{2DS}	H^2_{B}	**H^2_{BRC} [95% CI]**	v_g [95% CI]	v_e [95% CI]
BaseDiameter	0.315	0.325	**0.330 [0.109, 0.534]**	4474.58 [66.18, 7520.93]	9091.61 [7093.66, 12854.98]
TransectCount	0.308	0.530	**0.556 [0.367, 0.773]**	67.04 [24.07, 98.96]	53.57 [34.83, 73.75]
TallestStem	0.822	0.824	**0.852 [0.794, 0.959]**	2849.73 [733.14, 4241.21]	493.17 [295.76, 684.56]
MaxCanopyHght	0.876	0.892	**0.909 [0.860, 1.004]**	3311.74 [1161.68, 4652.61]	330.16 [203.25, 456.19]
StemDiameter	0.710	0.710	**0.710 [0.601, 0.857]**	1.80 [0.71, 2.59]	0.74 [0.57, 1.01]
Moisture	0.158	0.187	**0.205 [0.000, 0.411]**	4.74 [-0.09, 9.21]*	18.36 [11.59, 23.36]
DryMatter	0.556	0.605	**0.679 [0.532, 0.868]**	71833.23 [21181.31, 113233.24]	33979.69 [19825.28, 46237.46]
DOYFS1	0.686	0.749	**0.749 [0.498, 1.155]**	415.85 [-452.91, 818.83]*	139.26 [31.67, 278.53]
AvgeSen	0.911	0.916	**0.916 [0.876, 0.965]**	0.46 [0.12, 0.70]	0.04 [0.03, 0.06]
ES.28	0.403	0.403	**0.430 [0.218, 0.632]**	0.33 [-0.01, 0.54]*	0.44 [0.28, 0.58]
ES.35	0.348	0.348	**0.473 [0.232, 0.673]**	0.35 [0.07, 0.56]	0.39 [0.27, 0.55]
ES.51	0.300	0.304	**0.338 [0.151, 0.585]**	0.27 [0.05, 0.49]	0.52 [0.24, 0.77]
ES.63	0.389	0.396	**0.399 [0.215, 0.587]**	0.22 [0.05, 0.38]	0.33 [0.25, 0.47]
ES.77	0.398	0.419	**0.495 [0.316, 0.727]**	0.25 [0.02, 0.44]	0.25 [0.15, 0.36]
ES.91	0.479	0.488	**0.511 [0.370, 0.773]**	0.24 [0.05, 0.39]	0.23 [0.14, 0.32]
ES.105	0.378	0.410	**0.463 [0.276, 0.675]**	0.23 [0.03, 0.38]	0.27 [0.17, 0.37]
ES.119	0.174	0.177	**0.192 [0.005, 0.382]**	0.36 [-0.21, 0.72]*	1.51 [0.96, 2.06]
ES.133	0.000	0.000	**0.000 [-0.165, 0.000]***	0.000 [0.00, 0.00]*	0.01 [0.01, 0.01]
ES1DOY	0.554	0.554	**0.606 [0.442, 0.794]**	249.44 [45.43, 385.04]	161.93 [96.03, 219.43]
ES2DOY	0.441	0.447	**0.460 [0.261, 0.695]**	222.32 [61.82, 355.04]	261.50 [194.83, 359.7]
ES3DOY	0.349	0.418	**0.450 [0.223, 0.683]**	47.40 [9.89, 79.04]	57.87 [32.84, 81.85]
ES4DOY	0.413	0.443	**0.443 [0.261, 0.732]**	19.75 [3.88, 32.76]	24.86 [15.02, 33.91]
ES5DOY	0.427	0.446	**0.446 [0.265, 0.679]**	41.79 [9.45, 66.13]	51.90 [37.56, 73.38]
CanHght.105	0.531	0.563	**0.585 [0.402, 0.788]**	27.01 [4.49, 40.70]	19.17 [11.39, 27.20]
CanHght.119	0.453	0.474	**0.532 [0.357, 0.750]**	81.04 [-15.76, 121.96]*	71.34 [47.27, 95.00]
CanHght.133	0.756	0.762	**0.826 [0.759, 0.951]**	113.43 [49.70, 158.57]	23.86 [12.26, 31.96]
CanHght.148	0.763	0.775	**0.805 [0.720, 0.975]**	272.17 [103.01, 387.44]	66.10 [48.90, 91.69]
CanHght.158	0.749	0.768	**0.784 [0.701, 0.939]**	422.28 [196.06, 621.09]	116.07 [89.13, 158.23]
CanHght.176	0.762	0.779	**0.798 [0.698, 0.924]**	708.57 [364.24, 985.85]	178.97 [101.34, 255.14]
CanHght.189	0.770	0.793	**0.813 [0.716, 0.942]**	957.73 [455.70, 1502.94]	219.85 [149.12, 312.56]
CanHght.203	0.811	0.835	**0.857 [0.792, 0.951]**	1228.98 [518.02, 1801.70]	205.68 [122.97, 286.04]
CanHght.217	0.828	0.853	**0.882 [0.824, 1.011]**	1704.50 [464.74, 2485.70]	227.18 [141.65, 320.80]
CanHght.231	0.856	0.873	**0.894 [0.845, 1.011]**	2442.35 [1304.12, 3537.93]	288.08 [152.06, 389.98]
CanHght.246	0.853	0.878	**0.886 [0.832, 0.975]**	2753.75 [1257.53, 4104.05]	353.23 [255.21, 496.71]
CanHght.258	0.867	0.883	**0.903 [0.857, 0.993]**	3233.66 [1624.89, 4853.75]	346.75 [209.07, 476.96]
CanHght.272	0.876	0.891	**0.909 [0.863, 0.996]**	3507.13 [2070.63, 5314.02]	352.29 [209.24, 492.28]
CanHght.286	0.878	0.896	**0.909 [0.860, 0.960]**	3839.30 [1326.69, 5708.78]	382.75 [271.62, 529.16]
CanHght.300	0.876	0.899	**0.914 [0.870, 0.979]**	3893.81 [993.36, 5574.25]	365.18 [211.47, 504.68]
CanHght.314	0.879	0.898	**0.913 [0.870, 0.987]**	3761.76 [1472.67, 5801.70]	359.01 [221.25, 490.30]
**Average (SD)**	**0.587 (0.248)**	**0.61 (0.245)**	**0.637 (0.243)**		

* v_g not significant or generated an R warning when used in a relevant statistical function.

Table S4. Broad sense heritabilities of all traits measured in *M. sacchariflorus* during 2009 based on mixed models with spatial corrections based on (1) a two-dimensional spline (H^2_{2DS}), (2) random block effects only (H^2_{B}) and (3) random block, row and column effects (H^2_{BRC}). The latter model (values in bold) was chosen for all subsequent analyses, and estimates of genetic (v_g) and residual error (v_e) variance, as well as genotypic best linear unbiased predictors (BLUPs), were obtained from that model. Model-based parametric bootstrap 95% confidence intervals (95% CI) are shown in brackets (see Materials and methods).

Trait	H^2_{2DS}	H^2_{B}	**H^2_{BRC} [95% CI]**	v_g [95% CI]	v_e [95% CI]
DOYFS1	0.951	0.951	**0.959 [0.917, 1.058]**	1955.71 [-13.59, 3501.85]	84.44 [20.77, 168.88]
AvgeSen	0.876	0.884	**0.884 [0.830, 0.968]**	0.30 [0.13, 0.44]	0.04 [0.03, 0.05]
BaseDiameter	0.451	0.451	**0.451 [0.242, 0.732]**	18061.04 [1974.88, 30380.47]	21954.72 [17419.59, 30749.34]
DryMatter	0.586	0.634	**0.680 [0.531, 0.869]**	164638.79 [62453.30, 249710.21]	77572.23 [44750.38, 99065.65]
MaxCanopyHght	0.881	0.897	**0.924 [0.889, 0.991]**	4146.40 [2115.25, 5833.92]	343.43 [193.23, 495.24]
Moisture	0.293	0.342	**0.619 [0.461, 0.884]**	28.38 [12.93, 44.66]	17.48 [10.38, 24.25]
StemDiameter	0.857	0.858	**0.885 [0.835, 0.980]**	2.43 [0.94, 3.55]	0.32 [0.17, 0.44]
TallestStem	0.575	0.623	**0.704 [0.582, 0.907]**	1833.10 [325.00, 2780.32]	769.39 [425.67, 1092.88]
TransectCount	0.707	0.717	**0.746 [0.646, 0.940]**	104.04 [58.45, 166.98]	35.50 [19.73, 46.46]
ES.40	0.000	0.000	**0.000 [-0.063, 0.000]***	0.00 [-0.01, 0.00]	0.08 [0.06, 0.12]
ES.55	0.365	0.365	**0.374 [0.167, 0.593]**	0.14 [0.01, 0.26]	0.24 [0.16, 0.33]
ES.68	0.350	0.350	**0.350 [0.132, 0.565]**	0.15 [0.01, 0.25]	0.27 [0.20, 0.35]
ES.82	0.186	0.201	**0.153 [-0.065, 0.305]***	0.08 [-0.02, 0.15]*	0.42 [0.25, 0.54]
ES.96	0.295	0.434	**0.535 [0.336, 0.823]**	0.43 [0.04, 0.66]	0.37 [0.21, 0.54]
ES.110	0.411	0.411	**0.411 [0.167, 0.620]**	0.03 [0.00, 0.05]	0.04 [0.03, 0.06]
ES1DOY	0.419	0.419	**0.432 [0.274, 0.635]**	65.57 [16.35, 103.32]	86.19 [59.79, 119.45]
ES2DOY	0.208	0.238	**0.230 [0.016, 0.400]**	38.06 [-1.12, 69.12]*	127.68 [91.24, 179.32]
ES3DOY	0.197	0.258	**0.235 [0.014, 0.464]**	12.58 [-3.10, 23.42]	41.02 [20.68, 57.56]
ES4DOY	0.270	0.370	**0.432 [0.253, 0.690]**	17.72 [1.09, 28.41]	23.32 [12.72, 31.99]
ES5DOY	0.000	0.000	**0.005 [-0.188, 0.011]***	0.01 [-0.34, 0.02]*	1.72 [0.95, 2.47]
CanHght.110	0.540	0.568	**0.568 [0.405, 0.776]**	62.54 [28.37, 95.20]	47.64 [35.98, 67.14]
CanHght.125	0.645	0.729	**0.767 [0.659, 0.928]**	55.20 [18.97, 82.78]	16.78 [12.35, 22.06]
CanHght.138	0.749	0.795	**0.847 [0.777, 0.955]**	107.16 [49.10, 156.88]	19.33 [9.05, 27.45]
CanHght.152	0.782	0.811	**0.859 [0.791, 1.005]**	335.86 [79.41, 478.30]	55.12 [27.71, 73.70]
CanHght.161	0.813	0.835	**0.868 [0.816, 0.963]**	577.39 [258.88, 907.78]	87.68 [58.09, 130.69]
CanHght.180	0.809	0.838	**0.883 [0.819, 0.983]**	1206.80 [423.99, 1717.98]	159.19 [88.84, 211.01]
CanHght.194	0.802	0.823	**0.855 [0.788, 0.980]**	1538.03 [864.85, 2387.81]	260.77 [148.18, 360.50]
CanHght.210	0.824	0.851	**0.882 [0.819, 0.974]**	2097.40 [888.91, 3063.68]	280.02 [135.87, 380.87]
CanHght.223	0.834	0.859	**0.892 [0.839, 1.019]**	2604.76 [840.32, 3762.84]	316.31 [196.66, 432.89]
CanHght.236	0.863	0.884	**0.913 [0.870, 0.960]**	3279.91 [1152.20, 4768.42]	313.33 [186.47, 452.29]
CanHght.252	0.865	0.891	**0.918 [0.880, 1.018]**	3557.15 [1524.87, 5142.33]	317.90 [202.35, 453.22]
CanHght.264	0.877	0.894	**0.930 [0.897, 0.995]**	3910.07 [1849.64, 5723.45]	296.51 [143.01, 407.13]
CanHght.278	0.853	0.864	**0.876 [0.812, 0.953]**	3919.22 [703.16, 5936.36]	556.68 [334.64, 751.17]
CanHght.293	0.877	0.892	**0.922 [0.888, 1.030]**	4327.22 [2107.05, 6389.84]	367.97 [196.17, 511.49]
**Average (SD)**	**0.589 (0.283)**	**0.616 (0.279)**	**0.647 (0.287)**		

* v_g not significant or generated an R warning when used in a relevant statistical function.

Table S5. Individual traits selected in the multiple linear regression (MLR) models shown as high-level summaries on Table 3. Tabulated are traits for early season canopy heights (CanHght.133 in 2008 and CanHght.138 in 2009), max canopy heights and dry matter yield for *M. sinensis* (sin) and *M. sacchariflorus* (sac) in 2008 and 2009. See Table 1 for descriptions of the traits shown. The models were built using the trait BLUP values and all terms in the final models were significantly different from 0 at the 5% level.

Trait name	Species (year)	Adjusted R^2	Traits in final MLR model
Early canopy height			
CanHght.133	Sin (2008)	0.39	BaseDiameter, ES.105, ES4DOY, TransectCount
CanHght.138	Sin (2009)	0.30	BaseDiameter, ES.40, ES.82, ES.110, ES5DOY
CanHght.133	Sac (2008)	0.76	ES1DOY, ES4DOY, StemDiameter
CanHght.138	Sac (2009)	0.64	ES.82, StemDiameter
Max canopy height	Sin (2008)	0.34	ES.63, ES.105, ES3DOY, StemDiameter
	Sin (2009)	0.44	AvgeSen, BaseDiameter, ES.68, StemDiameter
	Sac (2008)	0.90	ES.51, ES1DOY, ES3DOY, StemDiameter
	Sac (2009)	0.87	StemDiameter
Dry matter yield	Sin (2008)	0.76	AvgeSen, BaseDiameter, CanHght.176, CanHght.314, TransectCount
	Sin (2009)	0.71	AvgeSen, CanHght.125, CanHght.161, CanHght.293, ES4DOY, MaxCanopyHght, Moisture, StemDiameter, TransectCount
	Sac (2008)	0.86	CanHght.148, CanHght.158, CanHght.272, TransectCount
	Sac (2009)	0.79	CanHght.138, CanHght.180, CanHght.223, MaxCanopyHght, TransectCount

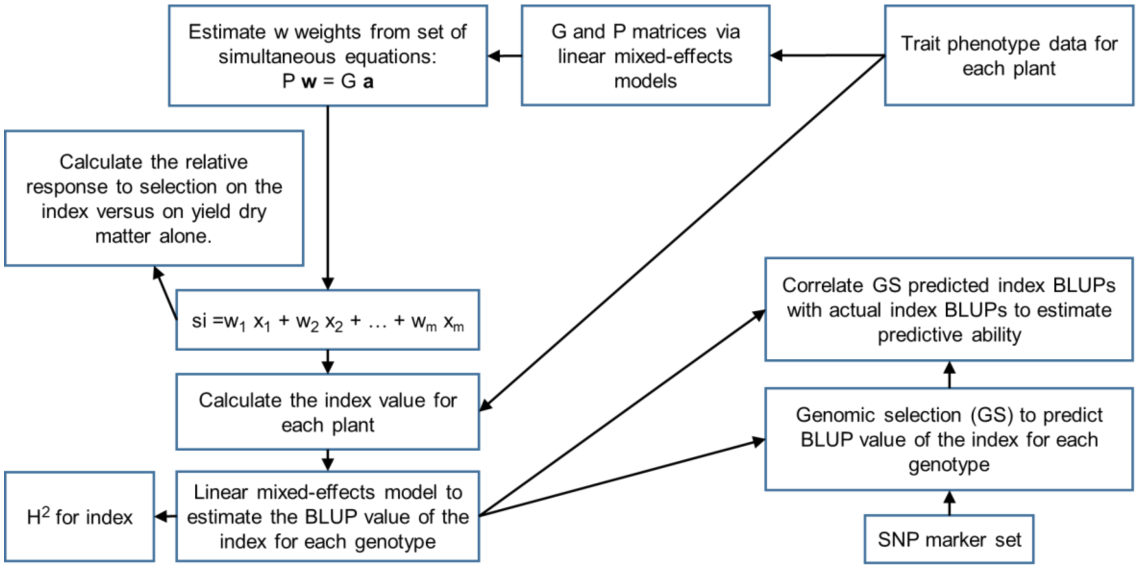

Figure S1. Flowchart of data analysis for the selection index calculations and subsequent genome-wide prediction (genomic selection). The selection index for dry matter yield (si) is calculated using Eqn. 4. The phenotypic values for each trait (e.g. x) are weighted by an equivalent "w" term which must be estimated for each trait in the equation. This is done by solving the matrix equation P **w**=G **a** for the vector "**w**" of "w" values. Vector "**a**" contains 0 apart from the first term which is 1 (equivalent to yield in the selection objective). The matrices G (genetic variance-covariance matrix) and P (phenotypic variance-covariance matrix) are populated with variances and covariances as described in the methods section using the genotypic and error variances estimated by linear mixed-effects models on the phenotypic data. Once the index equation has been defined then the index value (si) for each plant can be calculated by inserting in its phenotypic values ("x"s) and the estimated "w" values. The by-genotype BLUP values for the index are then estimated by applying a linear mixed-effects model to the plant index values. The genotypic and error variances then give the broad sense heritability (H^2) of the index. Genomic selection is then used to predict the index BLUP values and these are correlated with the actual index BLUP values to estimate the mean predictive abilities.
